# Multivariate patterns among multimodal neuroimaging and clinical, cognitive, and daily functioning characteristics in bipolar disorder

**DOI:** 10.1038/s41386-024-02047-2

**Published:** 2025-01-09

**Authors:** Viktoria Damgaard, Lydia Fortea, Johanna M. Schandorff, Julian Macoveanu, Bethany Little, Peter Gallagher, Gitte M. Knudsen, Lars V. Kessing, Kamilla W. Miskowiak

**Affiliations:** 1 Neurocognition and Emotion in Affective Disorders (NEAD) Centre, Psychiatric Centre Copenhagen, Mental Health Services, Capital Region of Denmark, Frederiksberg, Denmark; 2 Department of Psychology, University of Copenhagen, Copenhagen, Denmark; 3 Institut d’Investigacions Biomèdiques August Pi I Sunyer (IDIBAPS), Fundació Clínic per la Recerca Biomèdica (FCRB), Barcelona, Spain; 4 Department of Medicine, Institute of Neuroscience, University of Barcelona, Barcelona, Spain; 5 Translational and Clinical Research Institute, Faculty of Medical Sciences, Newcastle University, Newcastle upon Tyne, United Kingdom; 6 CNNP Lab, Interdisciplinary Computing and Complex BioSystems Group, School of Computing, Newcastle University, Newcastle upon Tyne, United Kingdom; 7 Neurobiology Research Unit and The Center for Experimental Medicine Neuropharmacology, Neurobiology Research Unit, Rigshospitalet, Copenhagen, Denmark; 8 Department of Clinical Medicine, University of Copenhagen, Copenhagen, Denmark; 9 Copenhagen Affective Disorder Research Centre (CADIC), Psychiatric Centre Copenhagen, Mental Health Services, Capital Region of Denmark, Frederiksberg, Denmark

**Keywords:** Cognitive neuroscience, Bipolar disorder

## Abstract

Individuals with bipolar disorder (BD) show heterogeneity in clinical, cognitive, and daily functioning characteristics, which challenges accurate diagnostics and optimal treatment. A key goal is to identify brain-based biomarkers that inform patient stratification and serve as treatment targets. The objective of the present study was to apply a data-driven, multivariate approach to quantify the relationship between multimodal imaging features and behavioral phenotypes in BD. We pooled structural, task and resting-state functional magnetic resonance imaging (MRI), and clinical, cognitive, and functioning data from 167 fully or partly remitted patients with BD from three studies conducted at the same site. We performed canonical correlation analysis (CCA) to investigate multivariate relations among the 56 imaging and 23 behavioral features in patients. Data from 46 matched healthy controls were included for covariate-adjusted standardization of patients’ scores and for group comparisons. The imaging and behavioral data sets showed a strong canonical correlation (*r* = 0.84, *p* = .004). Among the behavioral variables, cognitive test scores across psychomotor speed, verbal memory, and verbal fluency were associated with the multimodal imaging variate comprising task activation within the dorsolateral prefrontal cortex and supramarginal gyrus, also when other clinical and daily functioning variables were considered. Task activation within the dorsal prefrontal and parietal cognitive control areas constitutes a potential pro-cognitive treatment target.

## Introduction

Bipolar disorder (BD) is a neuropsychiatric disorder characterized by recurrent episodes of depression and (hypo)mania [1], which rank among leading causes of disability worldwide [2]. Patients with BD show considerable heterogeneity within clinical symptomatology, cognitive status, and daily functioning [3–6], which poses challenges for accurate diagnostics and providing optimized treatment. A crucial goal is therefore to identify brain-based biomarkers that provide insight into the underlying pathophysiology,—and how this may differ across subgroups with different phenotypic presentation—thereby informing patient stratification and serving as treatment targets [7, 8].

Neuroimaging studies in BD suggest that patients’ phenotypic heterogeneity can be detected at the underlying neurobiological level. Specifically, studies using data-driven approaches to identify cognitive subgroups in BD have shown evidence of aberrant task-related activation and functional connectivity within frontal-parietal cortices as well as reduced prefrontal cortical thickness and lower white matter volume in cognitively impaired patients [9–12]. Moreover, two studies—in which one has a partially overlapping sample with the current data - have demonstrated distinct BD subgroups based on neural activation during emotion regulation, which were characterized by differential activation across the prefrontal cortex and amygdala [13, 14]. Together, these findings suggest a link between structural and functional brain alterations and clinical and cognitive phenotypes in BD that could act as potential treatment targets.

A key methodological challenge in these studies examining brain-behavior associations is the conventional use of univariate approaches that assess a single measurement from one modality at a time [15, 16]. However, given the intercommunicative nature of the central nervous system, it is unlikely that one single brain area underpins complex and multifaceted phenotypes. Based on this, there is a need for multivariate approaches that can uncover patterns of brain-behavior associations among multiple measurements and modalities at a time [15]. One such technique is canonical correlation analysis (CCA), which enables investigation of covariation among two high-dimensional sets of variables without multiple correction steps [15, 17]. Despite the increased interest in CCA for brain-behavior associations, there are only few studies in BD. Of these, one explored multivariate associations among cognition and brain structure in 56 patients in remission [18]. Here, deficits across psychomotor speed, executive functioning, and verbal memory were associated with aberrant frontotemporal grey matter volume and temporal cortical thickness. Another study applied sparse CCA to investigate associations among multimodal imaging variables and behavior across 40 patients with BD, 100 patients with schizophrenia, and 50 healthy controls (HC) [19]. Specifically, age, IQ, and BMI were most strongly associated with multimodal imaging variables such as cortical thickness across frontal, parietal, and temporal cortices, working memory task-related prefrontal and parietal activity, and white matter fractional anisotropy [19]. Nevertheless, no published study to date has applied multivariate CCA to examine associations among multimodal neuroimaging and behavioral phenotypes specifically in BD.

The objective of the present study was therefore to perform CCA to explore multivariate relations among structural and functional neuroimaging variables and comprehensive measures of clinical features, cognition, and daily functioning in a large cohort of remitted patients with BD.

## Materials and methods

### Participants and procedure

Baseline behavioral and MRI data were pooled from three prior studies conducted at the same study site from January 2016 to June 2021: the Bipolar Illness Onset study [20], and the Prefrontal Target Engagement as a biomarker model for Cognitive improvement (PRETEC) studies [21, 22]. This yielded a sample of 167 patients with BD. Pooling data was appropriate given the overlapping inclusion criteria, neurocognitive tests, ratings of daily functioning and mood symptoms, MRI acquisition, and fMRI paradigm, as well as equal administration of these. All patients had an ICD-10 diagnosis of BD, which was confirmed with the Schedules for Clinical Assessment in Neuropsychiatry (SCAN) [23] interview by trained PhD students or postdoctoral researchers. We also included behavioral and MRI data from 46 HC with no history of treatment-requiring psychiatric disorder that were matched on age, sex, and IQ [20] in order to standardize patients’ behavioral and imaging scores and to identify fMRI task activation peaks. On the day of assessment, participants underwent neurocognitive testing, ratings of daily functioning and mood symptoms, and, within 0-3 days, an MRI scan.

Recruitment procedures and exclusion criteria for participants are available from ClinicalTrials.gov (BIO: NCT02888262; PRETEC-ABC: NCT03295305; PRETEC-EPO: NCT03315897). All studies have been approved by the Danish Research Ethics Committee for the Capital Region of Denmark (BIO: H-72014007; PRETEC-EPO: H-16043370; PRETEC-ABC: H-16043480) and The Danish Data Protection Agency Capital Region of Denmark (BIO: RHP-2015-023; PRETEC: 2012-58-0004). Written informed consent was obtained for all participants prior to participation in the respective studies.

### Behavioral data set

The behavioral data set contained 23 variables (see Supplementary Table S2 for detailed overview and definitions of each variable in the behavioral data set) and included the following clinical information: illness duration in years, number of prior depressive, (hypo)manic, and mixed episodes, and current depressive and (hypo)manic subsyndromal symptoms assessed by the Hamilton Depression Rating Scale — 17 Items (HDRS) [24] and Young Mania Rating Scale (YMRS) [25], respectively. Two daily functioning variables were included in the data set: total scores on the Functioning Assessment Short Test (FAST) scale [26] and the Work and Social Adjustment Scale (WSAS) [27]. The FAST is an observer-rated, interview-based scale that evaluates six domains of functioning: autonomy, occupational functioning, cognitive functioning, financial issues, interpersonal relationships, and leisure time. The WSAS is a self-report scale adressing social and occupational-related impairment. Finally, 15 cognitive test score variables were derived from the following neuropsychological test battery, covering the broad domains of attention, processing speed, executive function, and working memory, and verbal learning and memory: Rey Auditory Verbal Learning Test (RAVLT) [28], Trail Making Test (TMT) Part A & B [29], The Repeatable Battery for the Assessment of Neuropsychological Status (RBANS) Coding & Digit Span [30], Wechsler Adult Intelligence Scale (WAIS)-III Letter-Number Sequencing [31], Verbal Fluency letters S & D [32], and the Rapid Visual Processing (RVP) and Spatial Working Memory (SWM) from CANTAB (Cambridge Cognition Ltd.).

### Multimodal imaging data set

We acquired structural, task-related, and resting-state functional MRI data using a 3 Tesla Siemens Prisma scanner and a 64-channel head-coil at the Copenhagen University Hospital, Rigshospitalet (all scans used the same MRI acquisition protocol, see details in the Supplementary material). After the imaging analyses described below, we identified 34 structural MRI, 12 task-related fMRI, and 10 resting-state fMRI variables. The final imaging data set thus comprised 56 variables of regional brain measures (see Supplementary Table S3 for detailed overview and definitions of each variable in the imaging data set).

#### Structural MRI analysis

We used the FreeSurfer analysis suite (version 7.3.2) default workflow to process T1-weighted images for all participants. Processing included intensity normalization, skull-stripping, transformation to Talairach space, and automatic segmentation to extract grey and white matter components. The computed cortical surface accuracy was controlled using the ENIGMA Cortical Quality Control Protocol 2.0. After manual corrections of any observed error in cortical reconstructions, we extracted grey matter cortical thickness for 68 cortical regions. Cortical thickness measures for left and right hemispheres were combined and averaged to reduce the number of variables for the subsequent analyses, yielding 34 cortical thickness variables.

#### fMRI task data pre-processing and subject-level analyses

We included a verbal working memory N-back task based on prior evidence that dysfunction in their task-related neurocircuitries is a core dimension in BD [33, 34] (see Supplementary material for description of the paradigm). Task-related fMRI data was processed using the FMRI Expert Analysis Tool (FEAT; version 6.0.5) in FSL (FSL; www.fmrib.ox.ac.uk/fsl). Pre-processing steps included non-brain removal, image realignment, spatial normalization to a Montreal Neurological Institute (MNI) template, and spatial smoothing with a 5 mm full-width-half-maximum Gaussian kernel. We applied a high-pass temporal filtering cut-off of 100 s, and we corrected for geometric distortions related to the B0 field based on the acquired B0 field map.

At the first level, the task was modelled as blocks using a general linear model (GLM) with four conditions: 0-back, 1-back, 2-back, and 3-back which were convolved with double-gamma hemodynamic response function with added temporal derivatives. These conditions were used to calculate a working memory-load contrast which reflected a linear increase or decrease in activation from 0- to 3-back conditions.

#### fMRI task data group-level analyses

To identify task-related activation clusters, we performed a whole brain group-level analysis in the HC sample only in FEAT with FMRIB’s Local Analysis of Mixed Effects (FLAME) as estimation method [35]. We used the linear contrast described above, assessing both BOLD response increase from 0- to 3-back and decrease from 3- to 0-back. The significance level for clusters were set at *p* = .05, corrected for multiple comparisons and with a cluster-forming threshold of *Z* = 3.1 (*p* = .001). We identified 11 working memory-related activation peaks in HC (Supplementary Table S1). For each activation peak, we constructed 8 mm spherical regions of interest (ROIs) centered around the MNI coordinates. In cases of overlapping ROIs within the same contrast, we selected the cluster with the highest peak *Z*-value. In addition, we constructed a left dorsolateral prefrontal cortex (DLPFC) ROI using a 10 mm sphere around MNI coordinates *x* = −44, *y* = 18, *z* = 22 for the verbal working memory N-back task based on prior reports [9, 36]. Across both patients and controls, we extracted the mean percentage BOLD signal change from these 12 ROIs for subsequent analyses.

#### Resting-state fMRI data pre-processing and subject-level analyses

Resting-state fMRI data was pre-processed using Harmonized Analysis of Functional MRI pipeline (HALFpipe, version 1.2.2 [37]), an extension of fMRIPrep [38] designed to provide a standardized workflow. Pre-processing steps included slice-timing correction, motion correction, intensity normalization, nuisance signal regression, and bandpass filtering (0.01–0.1 Hz). The data were then registered to the individual T1-weighted image, normalized to the MNI space, and smoothed using a Gaussian kernel with a full-width-half-maximum of 6 mm. We applied the component-based noise correction method (CompCor [39]) to estimate and regress out noise components caused by physiological fluctuations, including signals of white matter and cerebral spinal fluid and head motion. Participants with an average relative framewise displacement >0.2 were excluded from further analysis.

To derive the spatial maps of the selected resting state networks (RSN) (i.e., the default mode network (DMN), salience network (SAL), and frontoparietal network (FPN)), we performed group-level independent component analysis (ICA) on the HC sample only using FSL’s tool of Exploratory Linear Optimized Decomposition into Independent Components (MELODIC), setting a maximum of 20 components [40]. The resulting components were then correlated with the seven-network parcellation from Yeo and colleagues [41] to identify the components with the highest correlation coefficient for each selected RSN. Based on visual inspection, we decided to further divide the FPN into two distinct networks corresponding to the right and left hemisphere due to its pronounced lateralization.

#### Resting-state fMRI data group-level analyses

After identifying the RSN components in the HC group, we used FSL’s dual regression [42] to extract the spatial and temporal maps from the participants. Dual regression consisted of two multivariate regressions: the first step extracted the subject-specific time-series of each component from the participant’s 4D resting-state fMRI dataset, and the second step derived subject-specific spatial maps (parameter estimate (PE) images) from the time-series. These subjective spatial maps indicated voxels connectivity within the ICA component representing an RSN while simultaneously controlling for influence from other ICA components [43]. After extracting the spatial and temporal time-series for each RSN, we calculated network integrity (within-network functional connectivity) and segregation (between-network functional connectivity) for each participant. RSN integrity was computed by averaging the PE across all voxels within each studied RSN. RSN segregation was assessed using the time-series from the first step of the dual regression where we calculated the Pearson’s correlation coefficients for each pair of RSNs and applied a Fisher’s transformation to standardize the values.

### Statistical analyses

Data normality distributions were explored using Shapiro-Wilk tests [44] and by visual inspection of histograms. To confirm group differences in demographics, clinical, and cognitive data in patients vs. HC, we conducted Pearson’s Chi-square (χ^2^) for dichotomous variables, independent samples *t*-tests for normally distributed continuous data, and Mann–Whitney *U* tests for non-parametric continuous data. We used R studio (version 2024.04.2+764) for statistical analyses with a set significance level of *p* < 0.05 (two-tailed).

Prior to analyses, behavioral raw scores and imaging values were standardized based on the mean and SD of the HC sample. However, for patients, we standardized the clinical and daily functioning variables (i.e., illness duration, number of mood episodes, HDRS and YMRS scores, and FAST and WSAS total scores) based on the mean and SD of the patient sample, as these variables had limited variability in HCs. Outliers (±3 SD) were further winsorized, and variables were reversed where appropriate so that a higher value reflects better performance/less impairment for interpretability purposes. As some participants had missing data in the behavioral data set (0.008% of data, see details in Supplementary material), we performed missing data imputation based on the available behavioral data with linear regression [45]. Imputation was performed separately for patients and HC samples as group differences were expected. To adjust for the effects of demographic variables on imaging and behavioral measures, we regressed out age and sex of the HC sample data using robust regression. Age and sex were then regressed out of the patient data based on the regression model estimated for the HC sample. The residuals for the patient data were then used for the subsequent multivariate analyses. Finally, key CCA assumptions of linearity and multivariate normality [17] were assessed by visual inspection of scatter plots and by Mardia’s tests of skewness and kurtosis (see more pre-processing details in the Supplementary material).

#### Canonical correlation analyses

To investigate multivariate associations among imaging and behavioral variables in patients, we performed CCA using the ‘*CCA’* package in R [46]. Briefly, CCA reduces the number of variables in each data set to latent variates that maximize the correlation (i.e., canonical correlation) between the two data sets. Other multivariate methods (e.g., partial least squares) were not considered for the present study, as it was primarily inspired by prior CCA studies with similar data sets in neuropsychiatric populations (e.g. [18, 19]).

Specifically, we conducted a global CCA including all imaging (*n* = 56) and behavioral (*n* = 23) variables. To assess whether the behavioral measures showed distinct associations with each imaging modality (i.e., cortical thickness, task-related activation, and resting-state functional connectivity), we also conducted ‘modular’ CCAs that included all behavioral features and the variables for each imaging modality, respectively.

The statistical significance of each of the obtained canonical correlations were determined with Wilks’ lambda tests [47] and verified with permutation testing [15, 17]. Here, the order of subjects (rows) is randomly shuffled, and the CCA is then performed 10,000 times on these mismatched data sets. The *p*-value represents the number of permutations that resulted in a higher correlation than the original model divided by the total number of permutations. The permutation test is thus corrected for multiple comparisons. We also assessed the stability of the obtained canonical correlations with 10-fold cross-validation [48]. Here, the data sets are separated into 10 folds, where one fold is randomly selected as test data and the remaining data (i.e., *K*-1) is used as training data. This cross-validation procedure is repeated systematically so that one fold is left out at a time until all folds have been used as test data. The mean and SD of the canonical correlation coefficients across folds are then compared to the original results.

For each statistically significant and stable model, we assessed the loadings and cross-loadings. Loadings represent the relation among a variable and the canonical variate of its *own* data set, whereas cross-loadings represent the association between a variable and the canonical variate of the *other* data set. Cross-loadings may thus more directly reflect which behavioral variables are associated with imaging measures. Loadings and cross-loadings that exceeded ±0.3 were considered substantial [16].

## Results

### Patients vs. healthy control comparisons

Table 1 shows the sample characteristics for patients and matched HC groups. As expected, patients had fewer years of education, poorer daily functioning, and poorer cognitive performance on 12 out of 15 tests compared to HC (*ps* < 0.003) (Supplementary Fig. S1). While patients had more depressive and (hypo)manic symptoms compared to HC (*ps* < 0.001), they were considered in full or partial remission at their time of assessment (patients’ HDRS: median = 4.0, IQR = 6.0; patients’ YMRS: median = 2.0, IQR = 4.0).

**Table 1:** Sample characteristics in patients with bipolar disorder and healthy controls.

	Patients	Controls
N	167	46
Sex (No. (%) female)	110 (66%)	30 (65%)
Age, median (IQR)	31.00 (15.00)	27.5 (31.00)
Verbal IQ, mean (SD)	111.38 (5.50)	112.55 (4.37)
Years of education, mean (SD)	14.00 (4.00)	16.00 (3.00)
HDRS, median (IQR)	5.00 (6.00)	1.00 (2.00)
YMRS, median (IQR)	2.00 (4.00)	0.00 (0.00)
Illness duration in years, median (IQR)	6.00 (8.00)	−
No. of depressive episodes, median (IQR)	6.00 (8.00)	–
No. of (hypo)manic episodes, median (IQR)	5.00 (8.00)	–
No. of mixed episodes, median (IQR)	0.00 (0.00)	–
Antidepressants (No. (%) yes)	42 (25%)	–
Antipsychotics (No. (%) yes)	58 (35%)	–
Anticonvulsants (No. (%) yes)	74 (44%)	–
Lithium (No. (%) yes)	79 (47%)	–
FAST total, median (IQR)	19.00 (18.50)	0.00 (2.00)
WSAS total, median (IQR)	20.00 (12.50)	0.00 (0.00)

*FAST* Functioning Assessment Short Test, *HDRS* Hamilton Depression Rating Scale, *IQR* Interquartile range, *SD* Standard deviation, *YMRS* Young Mania Rating Scale, *WSAS* Work and Social Adjustment Scale.

### Canonical correlation analysis

**Fig. 1:**
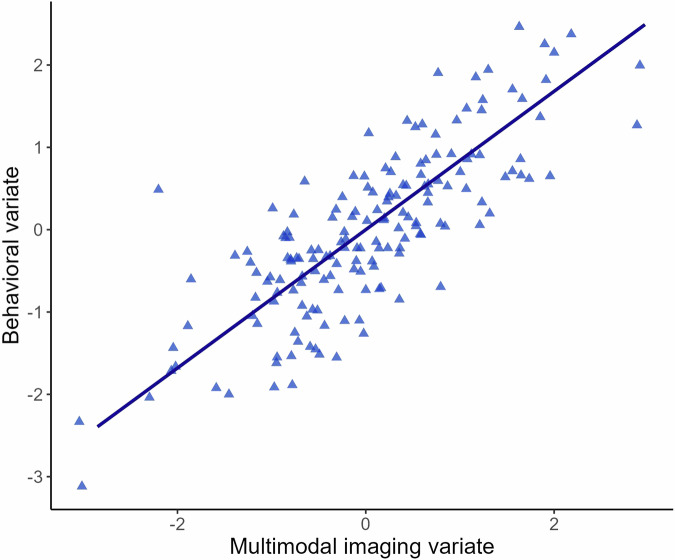
Global canonical correlation among imaging and behavioral variates. Scatterplot of the canonical correlation (*r* = 0.84) among the multimodal imaging and behavioral variates in patients with bipolar disorder.

**Fig. 2:**
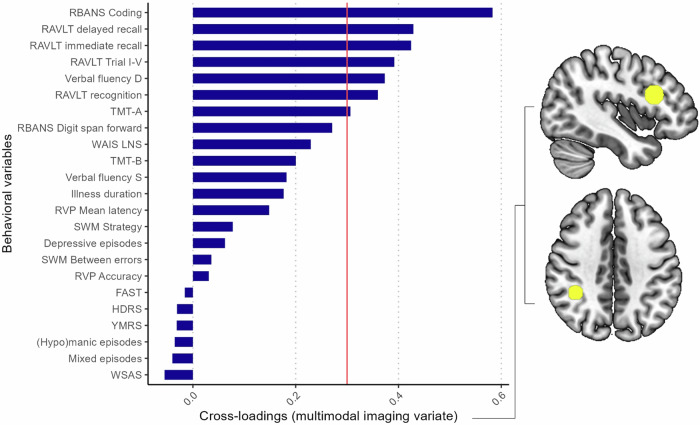
Cross-loadings for each behavioral variable on to the multimodal imaging variate. A cross-loading exceeding ±0.3 is considered substantial (threshold marked with red line). Cognitive test scores across psychomotor speed, verbal memory, and verbal fluency were associated with the multimodal imaging variate (cross-loadings: RBANS Coding [0.58], RAVLT Delayed [0.43], RAVLT Trial VI [0.42], RAVLT Trial I-V [0.39], Verbal Fluency D [0.37], RAVLT Recognition [0.36], and TMT-A [0.31]). The multimodal imaging variate comprised task activation within the left DLPFC (top right) and right supramarginal gyrus (bottom right).

For the global CCA containing all imaging modalities, the first canonical correlation was statistically significant (*r* = 0.84, *p* = .004; Fig. 1), which was verified with the permutation test (*p* = .004). The other variate pairs were not statistically significant (*ps* ≥ .09). The cross-validation procedure revealed that the canonical correlation was stable across folds (mean *r* = 0.87, SD = 0.01; see Supplementary Table S5), indicating that the obtained canonical correlation was not dependent on the subset of the sample. The multimodal imaging variate comprised fMRI task activation within the right supramarginal gyrus (loading=0.33) and left DLPFC (loading=0.31). Figure 2 shows the cross-loadings for each of the behavioral variables. Among the behavioral variables, cognitive test scores across psychomotor speed, verbal memory, and verbal fluency were associated with the multimodal imaging variate (cross-loadings: RBANS Coding [0.58], RAVLT Delayed [0.43], RAVLT Trial VI [0.42], RAVLT Trial I-V [0.39], Verbal Fluency D [0.37], RAVLT Recognition [0.36], and TMT-A [0.31], respectively). The full list of cross-loadings for each behavioral variable is provided in Supplementary Table S4.

#### Modular CCAs

For the sMRI CCA model (i.e., imaging data set including only structural brain variables), there was a statistically significant canonical correlation among the first variate pair (*r* = 0.76, *p* = 0.01), which was verified with the permutation test (*p* = 0.01). The cross-validation procedure confirmed that the correlation was stable (mean *r* = 0.78, SD = 0.01; Supplementary Table S5). The sMRI imaging variate comprised cortical thickness within the superior temporal lobe (loading = −0.32) and isthmus cingulate (loading = 0.32). Among the behavioral variables, cognitive test scores across psychomotor speed, verbal memory, and working memory were associated with the sMRI variate (cross-loadings: RAVLT Trial I-V [−0.42], RAVLT Delayed [−0.41], WAIS LNS [−0.41], RBANS Coding [−0.40], RAVLT Trial VI [−0.39], TMT-A [−0.37], and RBANS Digit-span [−0.31]) (Supplementary Fig. S2; full list of cross-loadings provided in Supplementary Table S4).

For the task fMRI CCA model (i.e., imaging data set including only task-related fMRI variables), there was a significant correlation among the first canonical variate pair (*r* = 0.63, *p* = 0.02), which was confirmed by the permutation test (*p* = 0.02). The cross-validation procedure showed that the obtained result was stable (mean *r* = 0.65, SD = 0.01; Supplementary Table S5). The task fMRI imaging variate consisted of activation within the following (pre)frontal and parietal regions: right supramarginal gyrus (loading = −0.79), left superior parietal lobe (loading = −0.71), left DLPFC (loading = −0.62), right superior parietal lobe (loading = −0.61), and right supramarginal gyrus (loading = −0.32). Among the behavioral variables, cognitive test scores across psychomotor speed, working memory, and executive functioning were associated with the task fMRI variate (cross-loadings: RBANS Coding [−0.42], TMT-B [−0.32], and SWM between errors [−0.32]) (Supplementary Fig. S3; full list of cross-loadings provided in Supplementary Table S4).

Finally, the resting-state functional connectivity CCA model did not show any statistically significant covariation between the resting-state and the behavioral data sets (*r* = 0.54, *p* = .35).

## Discussion

This is the first study to examine multivariate patterns among multimodal (structural and functional) brain imaging measures and clinical, cognitive, and daily functioning features in fully or partly remitted patients with BD. The global CCA revealed a strong and statistically significant pattern of covariance between the imaging and behavioral variables. Among the behavioral variables, cognitive tests assessing psychomotor speed, verbal memory, and verbal fluency were positively associated with the multimodal imaging variate, which comprised task activation within the left DLPFC and right supramarginal gyrus. Moreover, poorer cognitive test scores across psychomotor speed, verbal memory, and working memory were most strongly related to the sMRI variate comprising lower cortical thickness in the superior temporal lobe and higher isthmus cingulate thickness. For the task fMRI CCA, cognitive test scores across psychomotor speed, working memory, and executive functioning were positively associated with task-related activation within prefrontal and parietal regions. There was not a statistically significant association between resting-state fMRI and behavior, consistent with findings from a prior CCA study in psychosis [19].

Our findings highlight the relationship between cognitive performance and multimodal neuroimaging features in BD, also after adjusting for age and sex and when other clinical and daily functioning variables are considered. This supports prior evidence of direct associations between cognitive impairment and alterations in brain structure [12, 18, 49], task-related neural activity [9, 10], and resting-state functional connectivity [11, 50] in BD. Among the multimodal imaging features, task activation in the left DLPFC and right supramarginal gyrus had the strongest loading on the imaging variate, suggesting that these two regions may be particularly important for behavior — and predominantly cognitive functioning — in BD. Accordingly, we also found that cognitive tests of psychomotor speed, working memory, and executive functioning were particularly associated with the task fMRI variate comprising task activation across wide-spread prefrontal and parietal regions. Specifically, *poorer* cognition was associated with *lower* task-related activation within these regions, which aligns with findings from prior univariate fMRI studies in both BD [9, 10, 34, 51] and schizophrenia [52, 53]. Together, this suggests that hypo-activation within these cognitive control neurocircuitries underlies cognitive impairment and may thus serve as targets for pro-cognitive interventions. Indeed, prior cognition intervention trials in BD have demonstrated that treatment-related normalization of prefrontal and parietal activity correlates with pro-cognitive effects across treatment modalities [54].

Moreover, cognitive performance across psychomotor speed, verbal memory, and working memory showed a negative association with the sMRI variate comprising *lower* cortical thickness within the superior temporal lobe and *higher* thickness in the isthmus cingulate cortex. Prior multivariate CCA studies have also shown associations among thinner temporal cortices and cognitive impairment in psychosis [55] and depression [56], indicating that this could be a *trans*diagnostic structural biomarker of cognitive impairment across neuropsychiatric conditions. Similarly, univariate studies in BD have generally shown that *reduced* cortical thinning correlate to cognitive impairment and worse illness outcome [57–60], suggesting that illness burden and progression may accompany excessive grey matter loss. However, we also found that *thicker* isthmus cingulate cortex was related to poorer cognitive performance. This aligns with findings from a prior CCA study in euthymic BD showing associations among *thicker* cortices and poorer psychomotor speed and attention [18]. It could be possible that thicker cortices may arise from aberrant neurodevelopment, e.g., increased grey matter due to faulty neuronal pruning [12, 57], and/or patients’ medication use, e.g., lithium [60, 61].

Strengths of the study include the multivariate approach to investigate patterns among imaging and behavioral variables across modalities. Another strength was that analyses were covariance-adjusted for age and sex based on HCs since these factors influence imaging features [19]. Moreover, the results and stability of the CCAs were assessed using permutation tests and cross-validation procedures in line with proposed guidelines for CCA applications in studies of brain-behavior associations [62]. However, this study has several limitations. Firstly, our sample size was only modest for CCA. It has been argued that CCA requires a sample size at least 20 times the number of variables, although others have stated that a sample size of 50 subjects is sufficient to reliably detect strong canonical correlations [18, 63]. It has previously been shown that a subject-to-variable ratio of approximately 1.5 is sufficient to obtain stability in cases of strong canonical correlations among data sets [64]. The present study had a subject-to-variable ratio above 2, which aligns with these latter guidelines, although our findings should be confirmed in a larger sample. Secondly, we analyzed previously published imaging and behavioral data, although the present study differs from these prior investigations that used univariate approaches to examine brain-behavior relations with one imaging modality at a time (e.g., 9, 50). Moreover, we had to combine and average cortical thickness measures from each hemisphere to reduce the number of variables for the CCA [62]. This made it difficult to ascertain whether structural brain-behavior relationships were specific to one hemisphere. Our findings should therefore be expanded and replicated in larger samples. Other clinically relevant variables, including number of hospitalizations and prior medication status, were not available for this patient cohort, which might have influenced imaging and cognitive features [65]. Finally, the cross-sectional nature of the study did not allow investigations of causal relationships among imaging and clinical, cognitive, and daily functioning phenotypes.

This is the first study to explore multivariate patterns among multimodal imaging and clinical, cognitive, and daily functioning features in a large cohort of fully or partly remitted patients with BD. We found that cognitive measures across psychomotor speed, verbal memory, and verbal fluency were most strongly associated with multimodal imaging measures. Task activation within cognitive control areas including the DLPFC may serve as neurocircuitry treatment targets for pro-cognitive interventions in BD. Future studies should expand the present results by investigating multivariate brain-behavior associations with additional imaging modalities in larger samples.

## Supplementary information

Supplementary material

Supplementary Table S2

Supplementary Table S3

## Data Availability

The data used and analyzed in the current study are available from the corresponding author upon reasonable request.
