# Supplementary material for: Multivariate patterns among multimodal neuroimaging and clinical, cognitive, and daily functioning characteristics in bipolar disorder
Source: Neuropsychopharmacology. 2025 Jan 9;50(6):976–82. doi: 10.1038/s41386-024-02047-2 (PMC12032351; doi:10.1038/s41386-024-02047-2)

**Supplementary material**

**MRI acquisition**

We acquired structural, task-related, and resting state functional MRI data using a 3 Tesla Siemens Prisma scanner and a 64-channel head-coil at the Copenhagen University Hospital, Rigshospitalet. Blood oxygen level dependent (BOLD) fMRI was acquired using a T2*-weighted gradient echo spiral echo-planar (EPI) sequence with an echo time (TE) of 30 ms, repetition time (TR) of 2 seconds, and flip angle of 90°. A total of 300 volumes were obtained for resting state and the working memory task (146 volumes for the memory encoding task), each containing 32 slices with a thickness of 3 mm with 25% gaps in-between, and a field of view (FOV) of 230×230 mm using a 64×64 grid. BOLD images were registered to T1-weighted structural images (TR = 1900 ms; TE = 2.58 ms; flip angle = 9°; distance factor = 50%; FOV=230×230 mm; slice thickness = 0.9 mm). We further acquired a standard B0 field map sequence with the same FOV and resolution as the sequence above (TR = 400 ms; TE = 7.38 ms; flip angle=60°) used for geometric distortions correction of the BOLD images. Image quality was ensured by visual inspection.

**Working memory fMRI paradigm**

The working memory fMRI task was a verbal letter-variant N-back task, where participants were instructed to respond when a letter stimulus matched the one occurring *N* (1, 2, or 3) steps back in the stimulus sequence. The task involved three levels of working memory load: 1-back, 2-back, and 3-back. It also included a sensorimotor control condition (0-back), which required participants to respond when seeing the letter X. Each task load was presented in four blocks in a fixed pseudo-random order after an instruction screen displayed for 12 seconds. Each block contained 10 letters, including three target stimuli shown for 0.5 seconds each with a fixed interstimulus interval of 1.5 seconds. Blocks were separated by a fixation cross for five seconds. The total task duration was nine minutes and 52 seconds.

**Data pre-processing and multivariate normality assumptions**

To avoid outliers skewing the CCA results, we performed outlier winzorization (±3 SD). Within the behavioral data sets, 18 out of 23 variables were affected: No. depressive episodes, No. (hypo)manic episodes, No. mixed episodes, YMRS total score, FAST total score, WSAS total score, RAVLT Trial I-V, RAVLT Trial VI, RAVLT Delayed, RAVLT Recognition, TMT-A, TMT-B, RBANS Coding, WAIS Letter-Number Sequencing, Verbal fluency S, RVP accuracy, RVP mean latency, SWM between errors. Notably, the patient data only contained values below 3 SD from HC. Within the imaging data, 43 out of 56 variables were affected, including all task fMRI, resting state fMRI [SAL within-network connectivity, left and right FPN within-network connectivity, right FPN- DMN between-network connectivity, left FPN-DMN between-network connectivity], and cortical thickness [caudal anterior cingulate thickness, caudal middle frontal thickness, cuneus thickness, fusiform thickness, inferior parietal cortex thickness, inferior temporal cortex thickness, lateral occipital cortex thickness, lateral orbitofrontal cortex thickness, lingual gyrus thickness, middle temporal gyrus thickness, parahippocampal gyrus thickness, paracentral cortex thickness, pars opercularis thickness, isthmus cingulate thickness, pars triangularis thickness, pericalcarine cortex thickness, rostral anterior cingulate cortex thickness, rostral middle frontal cortex thickness, superior frontal gyrus thickness, superior temporal gyrus thickness, supramarginal gyrus thickness, frontal pole thickness, posterior cingulate cortex thickness, precentral gyrus thickness, transverse temporal thickness, and temporal pole thickness] variables, respectively.

Some participants had missing data for the behavioral variables. Specifically, 0.008% of the behavioral data was missing (patients=0.008%; HC=0.005%). The variables with missing data were: WSAS total scores (0.05% missing), No. of depressive episodes (0.02% missing), No. of (hypo)manic episodes (0.02% missing), No. of mixed episodes (0.05% missing), RAVLT Trial I-V (0.005% missing), RAVLT Trial VI (0.005% missing), RAVLT Delayed (0.005% missing), RAVLT Recognition (0.005%), Verbal fluency S (0.005% missing), Verbal fluency D (0.005% missing), RVP accuracy (0.005% missing), and RVP mean latency (0.005% missing). The missing data was considered ‘missing at random’ as it was randomly distributed across behavioral variables. We therefore performed missing data imputation based on the available behavioral data with linear regression. Imputation was performed separately for patients and HC samples as group differences were expected.

The Mardia’s test revealed that the imaging data set had multivariate normality (skewness: *p*≥.07, kurtosis: *p*≥.50), but not the behavioral data set *(ps*≥.001 for skewness and kurtosis). We then inspected individual variables within the behavioral data set and transformed skewed variables as required to meet assumptions of multivariate normality. Specifically, number of depressive, (hypo)manic, and mixed episodes, illness duration, and YMRS and HDRS scores were transformed using log or square root transformation where appropriate. The remaining variables did not require transformation. After transformation, the behavioral data set had multivariate normality according to the Mardia’s tests *(*skewness: *p*=.61, kurtosis: *p*=.06). We also performed the CCA on the untransformed behavioral data (i.e., without individual variable transformation), which did not change the results.

**Supplementary Table S1:** Clusters showing task-related activation in healthy controls during the verbal N-back working memory task.

|  |  |  |  |  | **MNI** | | |
| --- | --- | --- | --- | --- | --- | --- | --- |
| **Anatomical region** | **BA** | ***P*-value** | **Cluster size (no. voxels)** | **Peak Z-value** | **X** | **Y** | **Z** |
| *Linear increase 0- to 3-back* |  |  |  |  |  |  |  |
| Right supramarginal gyrus, posterior division | 40 | <.001 | 73594 | 10.6 | 38 | -44 | 42 |
| Right superior parietal lobule | 7 |  |  | 10.3 | 34 | -60 | 48 |
| Right supramarginal gyrus, posterior division | 40 |  |  | 10.2 | 52 | -44 | 52 |
| Left superior parietal lobule | 40 |  |  | 10.2 | -34 | -54 | 42 |
| *Linear decrease 0- to 3-back* |  |  |  |  |  |  |  |
| Precuneous cortex | 30 | <.001 | 48039 | 10 | -6 | -52 | 10 |
| Right frontal pole | 9 | <.001 | 209 | 6.05 | 16 | 56 | 46 |
| Right parietal operculum cortex | 48 |  |  | 9.69 | 50 | -26 | 20 |
| Left central opercular cortex | 48 |  |  | 9.31 | -38 | 2 | 12 |
| Left medial precentral gyrus | 31 |  |  | 9.25 | -12 | -34 | 44 |
| Right central opercular cortex | 48 |  |  | 9.23 | 50 | 0 | 8 |
| Left parahippocampal gyrus, posterior division | 37 |  |  | 9.08 | -28 | -34 | -16 |

Abbreviations: BA=Broadman area; MNI=Montreal Neurological Institute**.**

**Supplementary Table S4.** Cross-loading values for each of the behavioral variables in the canonical correlation analyses showing statistically significant covariation between imaging and behavioral data sets (both global and modular imaging models). A cross-loading of ±0.3 are considered meaningful.

|  | **Global CCA** | **sMRI CCA** | **Task fMRI CCA** |
| --- | --- | --- | --- |
| HDRS | -0.03 | -0.22 | -0.02 |
| YMRS | -0.03 | 0.04 | -0.01 |
| FAST | -0.02 | -0.13 | -0.07 |
| WSAS | -0.06 | -0.11 | -0.08 |
| No. depressive episodes | 0.06 | -0.05 | -0.07 |
| No. (hypo)manic episodes | -0.04 | 0.04 | 0.09 |
| No. mixed episodes | -0.04 | 0.02 | 0.12 |
| RAVLT Trial I-V | **0.39** | **-0.42** | -0.21 |
| RAVLT Trial VI | **0.42** | **-0.39** | -0.29 |
| RAVLT Delayed | **0.43** | **-0.41** | -0.23 |
| RAVLT Recognition | **0.36** | -0.28 | -0.20 |
| TMT-A | **0.31** | **-0.37** | -0.18 |
| TMT-B | 0.20 | -0.15 | **-0.32** |
| RBANS Coding | **0.58** | **-0.40** | **-0.42** |
| RBANS Digit-span | 0.27 | **-0.31** | -0.27 |
| WAIS LNS | 0.23 | **-0.41** | -0.06 |
| Verbal fluency S | 0.18 | 0.07 | -0.18 |
| Verbal fluency D | **0.37** | -0.11 | -0.25 |
| RVP A | 0.03 | -0.08 | -0.23 |
| RVP mean latency | 0.15 | -0.04 | -0.04 |
| SWM between errors | 0.04 | 0.02 | **-0.32** |
| SWM strategy | 0.08 | 0.03 | -0.21 |
| Illness duration | 0.18 | -0.16 | -0.09 |

Abbreviations: CCA=Canonical correlation analysis; FAST=Functioning Assessment Short Test; fMRI=Functional magnetic resonance imaging; HDRS=Hamilton Depression Rating Scale – 17 items; LNS=Letter-Number Sequencing; RAVLT=Rey Auditory Verbal Learning Test; RBANS=Repeated Battery for the Assessment of Neuropsychological Status; RVP=Repeated Visual Processing; sMRI=Structural magnetic resonance imaging; SWM=Spatial Working Memory; TMT=Trail Making Test; WAIS=Wechsler Adult Intelligence Scale; WSAS=Work and Social Adjustment Scale; YMRS=Young Mania Rating Scale.

**Supplementary Table S5.** Results from the K-fold cross validation procedure. The obtained first canonical correlation for each fold for the global and modular imaging CCAs showing statistically significant covariation between imaging and behavioral data sets.

|  | **Global CCA** | **sMRI CCA** | **Task fMRI CCA** |
| --- | --- | --- | --- |
| Mean across folds | 0.87 | 0.78 | 0.65 |
| SD | 0.01 | 0.01 | 0.01 |
| Fold #1 | 0.87 | 0.77 | 0.66 |
| Fold #2 | 0.86 | 0.79 | 0.65 |
| Fold #3 | 0.87 | 0.80 | 0.64 |
| Fold #4 | 0.87 | 0.77 | 0.65 |
| Fold #5 | 0.85 | 0.78 | 0.64 |
| Fold #6 | 0.87 | 0.78 | 0.64 |
| Fold #7 | 0.88 | 0.78 | 0.66 |
| Fold #8 | 0.89 | 0.77 | 0.64 |
| Fold #9 | 0.86 | 0.76 | 0.65 |
| Fold #10 | 0.86 | 0.78 | 0.66 |

Abbreviations: CCA=Canonical correlation analysis; fMRI=Functional magnetic resonance imaging; sMRI=Structural magnetic resonance imaging.

**Supplementary Figure S1:** Cognitive test scores in patients with bipolar disorder vs. healthy controls.


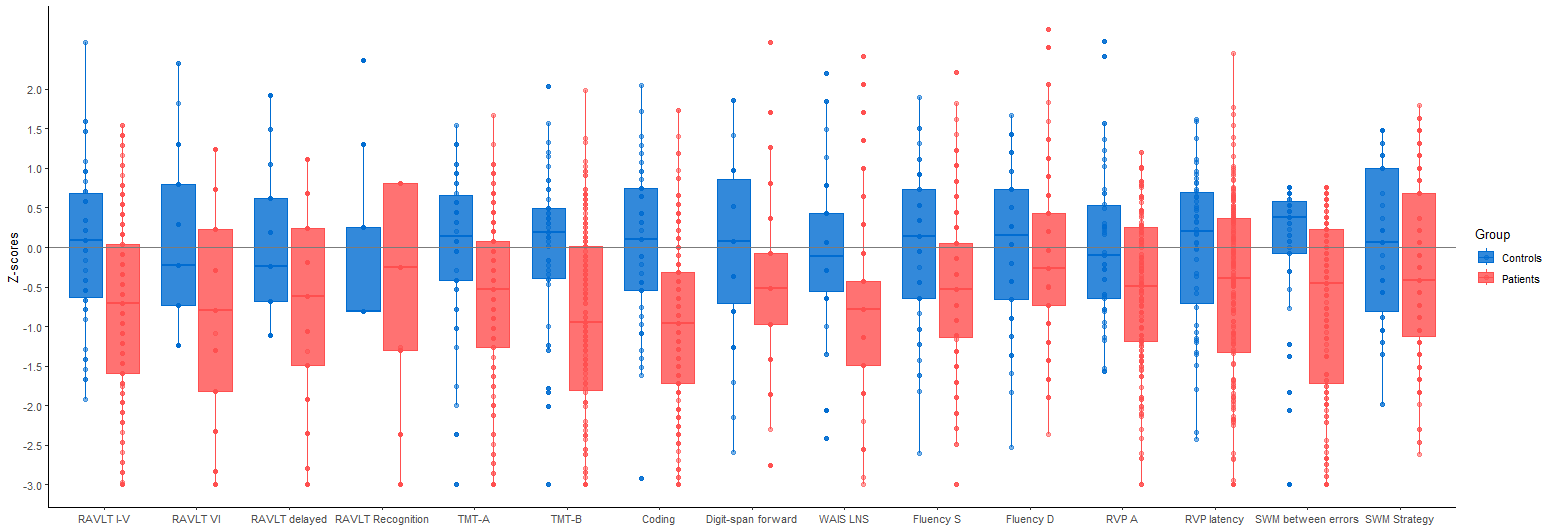


Abbreviations: LNS=Letter-Number Sequencing; RAVLT=Rey Auditory Verbal Learning Test; RVP=Rapid Visual Processing; SWM=Spatial Working Memory; TMT=Trail Making Test; WAIS=Wechsler Adult Intelligence Scale.

RAVLT Trial I-V total recall (*p*<.001), RAVLT VI recall (*p*<.001), RAVLT delayed recall (*p*=.003), RAVLT Recognition (*p*=.11), TMT part A (*p*<.001), TMT part B (*p*<.001), Coding (*p*<.001), Digit-Span forward score (*p*<.001), WAIS LNS (*p*<.001), Verbal fluency S (*p*=.003), Verbal fluency D (*p*=.31), RVP Accuracy (*p*=.001), RVP mean latency (*p*=.005), SWM between errors (*p*<.001), SWM strategy (*p*=.07). Unadjusted p-values.

**Supplementary Figure S2:** Cross-loadings for each behavioral variable on the structural imaging variate. The structural imaging variate comprised lower cortical thickness in the superior temporal lobe and higher isthmus cingulate cortex thickness.

**
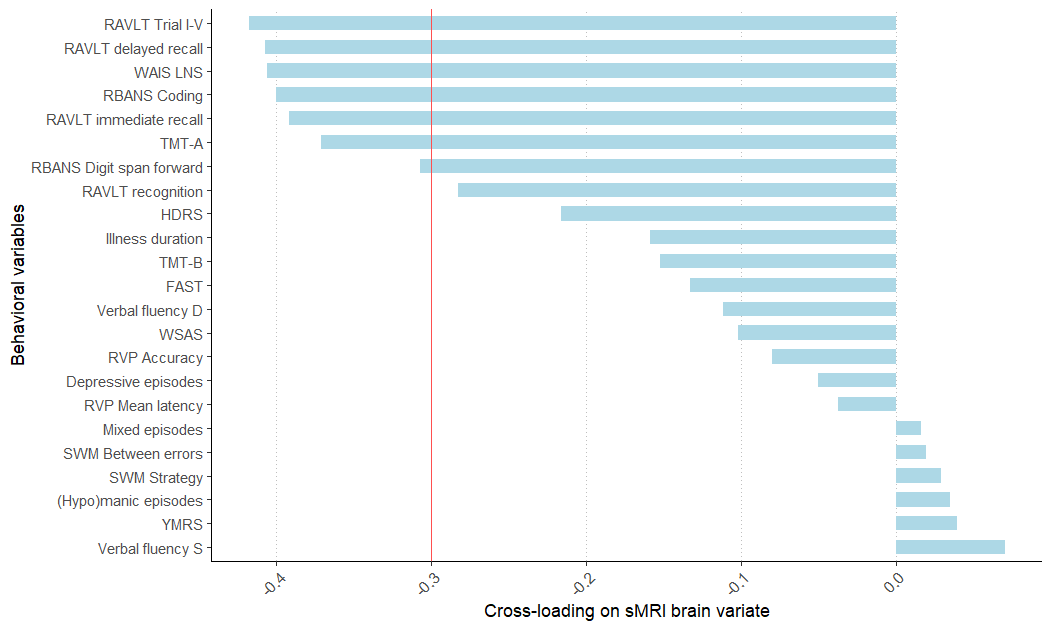
**

**Supplementary Figure S3:** Cross-loadings for each behavioral variable on the task-related fMRI variate. The task fMRI variate consisted of hypo-activation within (pre)frontal and parietal regions during a working memory task.


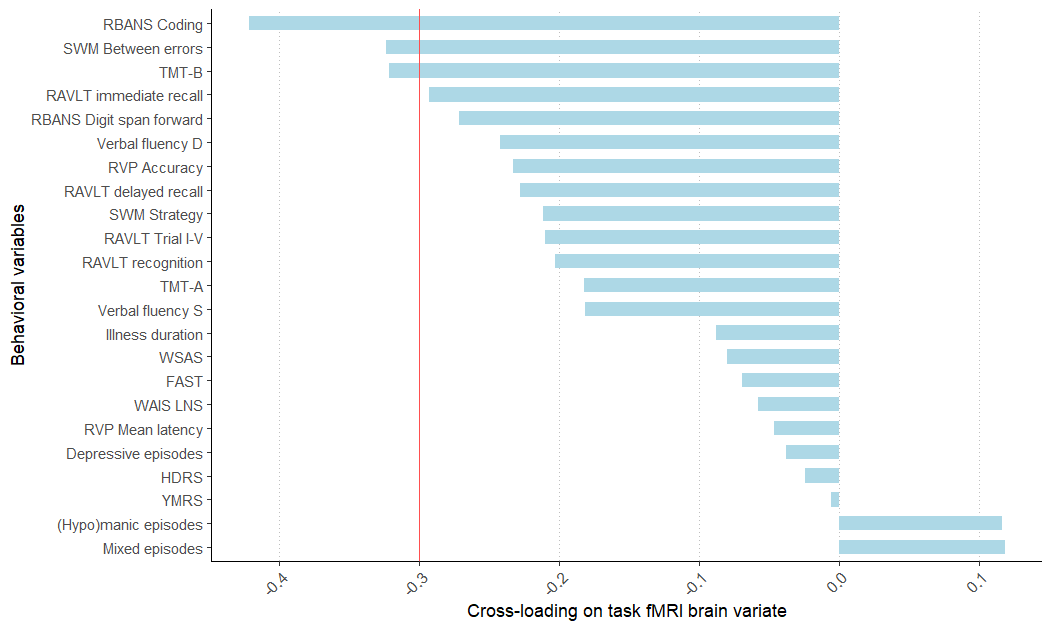

Supplement: Supplementary file 1 — Supplementary material [file 41386_2024_2047_MOESM1_ESM.docx]
